# Recent Developments for Remediating Acidic Mine Waters Using Sulfidogenic Bacteria

**DOI:** 10.1155/2017/7256582

**Published:** 2017-10-03

**Authors:** Ivan Nancucheo, José A. P. Bitencourt, Prafulla K. Sahoo, Joner Oliveira Alves, José O. Siqueira, Guilherme Oliveira

**Affiliations:** ^1^Facultad de Ingeniería y Tecnología, Universidad San Sebastián, Lientur 1457, 4080871 Concepción, Chile; ^2^Instituto Tecnológico Vale, Rua Boaventura da Silva 955, 66055-090 Belém, PA, Brazil; ^3^SENAI Innovation Institute for Mineral Technologies, Av. Com. Brás de Aguiar 548, 66035-405 Belém, PA, Brazil

## Abstract

Acidic mine drainage (AMD) is regarded as a pollutant and considered as potential source of valuable metals. With diminishing metal resources and ever-increasing demand on industry, recovering AMD metals is a sustainable initiative, despite facing major challenges. AMD refers to effluents draining from abandoned mines and mine wastes usually highly acidic that contain a variety of dissolved metals (Fe, Mn, Cu, Ni, and Zn) in much greater concentration than what is found in natural water bodies. There are numerous remediation treatments including chemical (lime treatment) or biological methods (aerobic wetlands and compost bioreactors) used for metal precipitation and removal from AMD. However, controlled biomineralization and selective recovering of metals using sulfidogenic bacteria are advantageous, reducing costs and environmental risks of sludge disposal. The increased understanding of the microbiology of acid-tolerant sulfidogenic bacteria will lead to the development of novel approaches to AMD treatment. We present and discuss several important recent approaches using low sulfidogenic bioreactors to both remediate and selectively recover metal sulfides from AMD. This work also highlights the efficiency and drawbacks of these types of treatments for metal recovery and points to future research for enhancing the use of novel acidophilic and acid-tolerant sulfidogenic microorganisms in AMD treatment.

## 1. Introduction

Metal mining provides everyday goods and services essential to society. However, this activity has at times caused extensive and sometimes severe pollution of air, vegetation, and water bodies [1]. Streams draining active or abandoned mines and mine spoils are widely considered as hazardous to human health and the environment, but on the other hand, they may also be alternative potential sources of valuable metals [2, 3].

Currently, millions of tons of ores are processed every year by the mining industry and are disposed in the form of waste rocks and mine tailings. As higher-grade ores are diminishing, the primary ores that are processed by mining companies are of increasingly lower grade (metal content) and the growing amount of waste material produced by mining operations is consequently significant. The use of lower grade ore was made possible by the development of the flotation technique in the late 19th century, which allowed the separation of metal sulfide minerals from gangue minerals that have no commercial value [4]. As a result of selective flotation, about 95 to 99% of the ground primary ores end up as fine-grain tailings, in the case of copper ores. The composition of tailings is directly dependent on that of the ore, and therefore they are highly variable, though pyrite (FeS_2_) is frequently the most reactive and dominant sulfide mineral present in tailings wastes [4–6].

**Figure 1:**
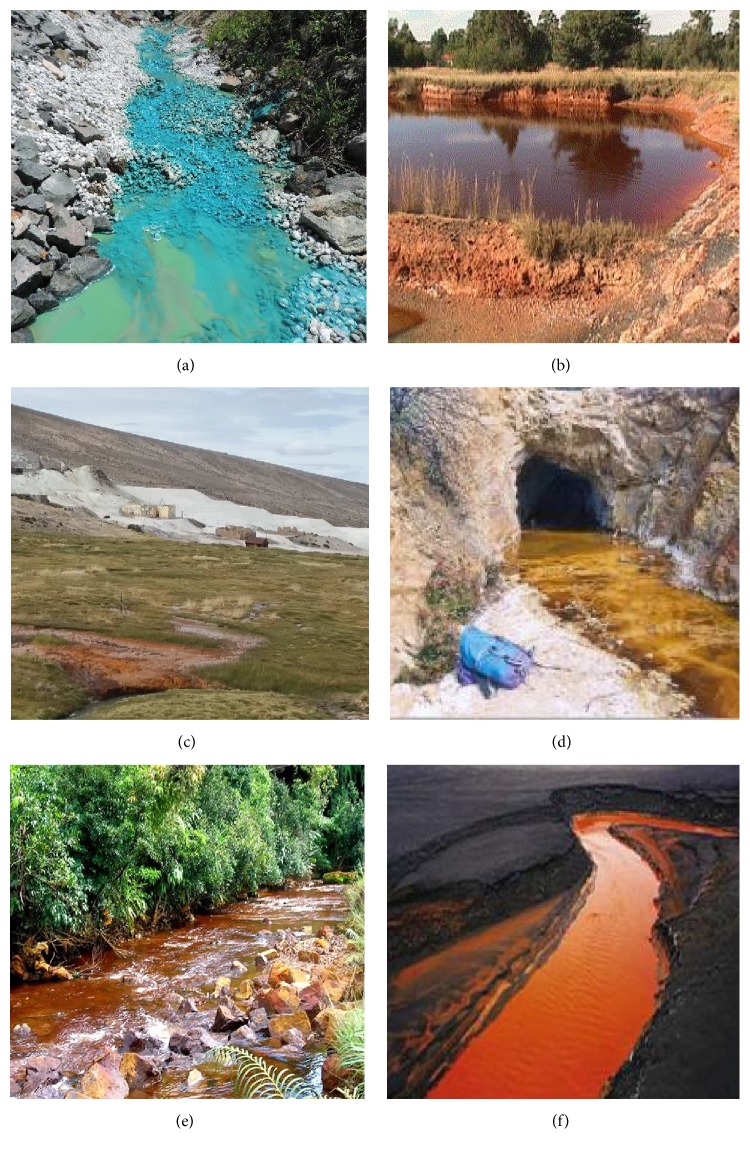
Illustration of streams of acidic waters draining from active or abandoned mines and mine spoils. (a) AMD from a copper mine in the State of Pará, Brazil, that has been remediated with limestone treatment, (b) acidic water released from abandoned underground metalliferous mine in the Republic of South Africa (reproduced from Akcil and Koldas [9]), (c) acidic mine water draining from an abandoned sulfur mine, northern Chile, (d) AMD discharge in the Lomero-Poyatos mine, Spain (reproduced from España et al. [10]), (e) acidic water draining from Coal mines, Jaintia Hills, and (f) AMD originated from mine tailings, Canada, (reproduced from Burtnyski [11]).

Pyritic mine tailings therefore have the potential to become extremely acidic when in contact with surface water. Under oxidizing conditions, pyrite-bearing wastes produce sulfuric acid. The acidic water further dissolves other metals contained in mine waste, resulting in low pH water enriched with soluble sulfate, Fe, Al, and other transition metals, known as acid mine drainage (AMD) (Figure 1) [7, 8].

## 2. Remediation of Acidic Mine Water

Waters draining from abandoned metal mines and mine wastes are often acidic (pH < 4) and contain elevated concentrations of dissolved metals and metalloids and high osmotic potential associated with concentration of sulfate salts [14]. In most cases, active chemical treatment and passive biological treatment can provide effective remediation of AMD [15] (details and literature of the advantages and disadvantages of these treatment and others are presented in Table 1). A major drawback to both approaches is that the immobilized metals are contained in “sludge” (chemical treatment) or within spent compost (biological treatment) and need to be disposed in specially designated landfill sites, precluding their recovery and recycling. Changes in redox conditions during storage can lead to remobilization of metals (and metalloids such as arsenic) in both sludge and spent composts. In addition, potentially useful and valuable metal resources are not recovered using conventional approaches for remediating mine waters [3, 16].

A radically different approach for remediating AMD which, like compost bioreactors, derives from the abilities of some microorganisms to generate alkalinity and to immobilize metals, is referred to generally as “active biological treatment.” Microbiological processes that generate alkalinity are mostly reductive processes and include denitrification, methanogenesis, and dissimilatory reduction of sulfate, ferric iron, and manganese (IV), which tend to be limited in AMD. Considering that AMD usually contains elevated concentrations of both ferric iron and sulfate, the ability of some bacteria to use these compounds as terminal electron acceptors suggests that these reactions can be highly useful for mine water remediation. Acidic environments in which sulfur or sulfide minerals are subjected to biologically-accelerated oxidative dissolution characteristically contain large concentrations of soluble sulfate [17]. Therefore, microbial sulfate reduction might be anticipated to occur within anaerobic zones in both acidic and nonacidic environments. Biological sulfidogenesis generates hydrogen sulfide as a result of a reductive metabolic process using sulfate reducing bacteria (SRB). Biological sulfidogenesis has the additional benefits of being a proton-consuming reaction, allowing the increase in pH of the mine water treated contributing towards mitigation and remediation. The hydrogen sulfide generated can be used in controlled situations to selectively precipitate many potentially toxic metals (such as copper and zinc) often present in AMD at elevated concentrations [3, 18]. Active biological treatment has many advantages over alternative strategies for treating mine waters, one of the most important being its potential for recovering metals that are commonly present in AMD.

**Figure 2:**
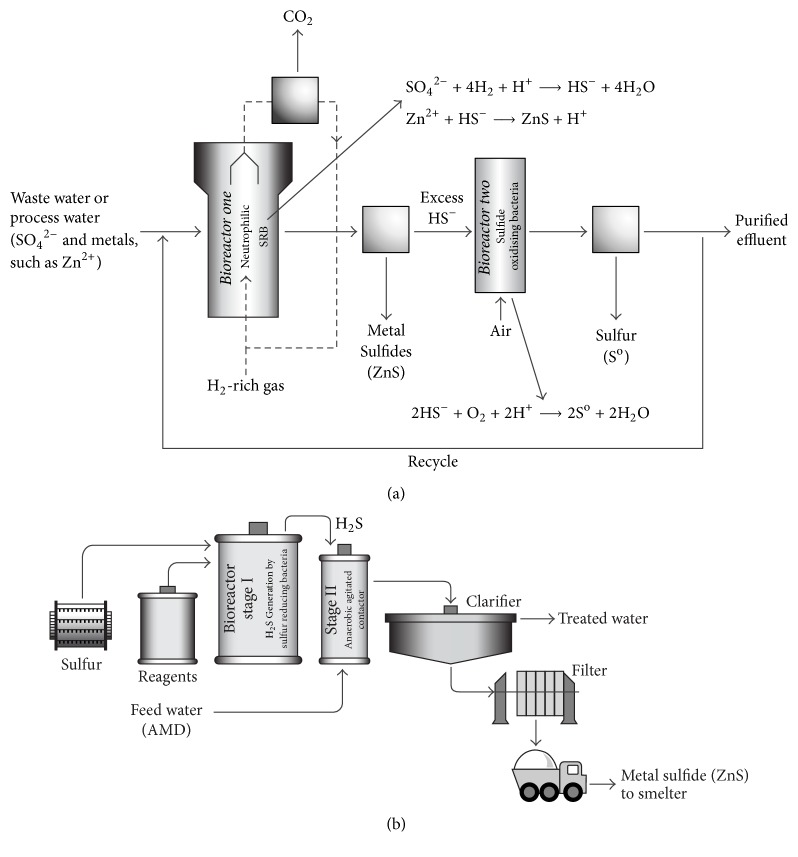
Schematic overview of the Thiopaq (a) and Biosulfide (b) processes (adapted from Adams et al. [12], Muyzer and Stams [13]).

There have been few successful applications of SRB-mediated active AMD treatment systems, even though this possibility has long been appreciated. One major reason for this is that SRB happens preferentially between pH 6 and 8 [19], whereas AMD generally has a pH between 2 and 4 and commonly pH < 3 [20]. Under these circumstances, a neutralization step is necessary before AMD effluents are subjected to bacterial sulfate reduction or, alternatively, “off-line” systems need to be used. The latter is necessary by the fact that current systems use neutrophilic SRB or sulfur reducing bacteria, and direct exposure to the inflowing acidic solution being treated would be lethal to these microorganisms. Therefore, a separate vessel in which sulfide generated by the bacteria is contacted with the acidic, metal-laden waste water, is required [16, 21]. Examples of this technology are the Biosulfide and Thiopaq processes (Figure 2) operated under the auspices of two biotechnology companies, BioTeq (Canada) and Paques B. V. (The Netherlands), which are currently in operation in various parts of the world.

The Biosulfide process has two stages, one chemical and the other biological. Metals are removed from AMD in the chemical stage by precipitation with biogenic sulfide produced in the biological stage by SRB under anaerobic condition. In this system, hydrogen sulfide is generated by the reduction of elemental sulfur, or other sulfur source, in the presence of an electron donor, such as acetic acid. The gas is passed to an anaerobic agitated contactor in which copper can be precipitated as a sulfide, usually without pH adjustment and without significant precipitation of other heavy metals present in the water. The end result is a high value copper product, usually containing more than 50% of the metal. Other metals such as nickel, zinc, and cobalt can also be recovered as separate high-grade sulfide products, although pH control using an alkali source is usually required to selectively precipitate the metal as a sulfide phase. The high-grade metal sulfide precipitate is then recovered by conventional clarification and filtration to produce a filter cake which can be shipped to a smelter [12].

The Thiopaq process uses another system that involves the use of two biological continuous reactors connected in series (I) to an anaerobic upflow sludge blanket (UASB) reactor for the reduction of oxidized sulfur species. In this reactor, ethanol or hydrogen is utilized by the SRB as electron donor, producing sulfide (mostly HS^−^) for the precipitation of metal sulfides (which can proceed in the same reactor depending on the toxicity of the wastewater), and (II) an aerobic submerged fixed film (SFF) reactor where the excess sulfide is oxidized to elemental sulfur, using sulfide-oxidizing bacteria. In this process, metals such as Zn and Cd can be precipitated down to very low concentrations [22].

The Paques B. V. process has been successfully implemented at an industrial scale at the gold mine Pueblo Viejo, located in the Dominican Republic. A copper recovery plant installed in 2014 based on sulfide precipitation is used to recover the copper liberated from the gold extraction process. The sulfidogenic bioreactor generates H_2_S to recover up to 12,000 ton of copper per year generating value and reducing the amount of copper sent to the tailing dam [23]. Application of this process has also been demonstrated on a pilot-scale at the Kennecott Bingham Canyon copper mine in Utah, where >99% of copper present in a pH 2.6 waste stream was recovered [22, 24, 25].

Sulfate reduction activity has been reported in low pH ecosystems, for example, in acidic lakes, wetlands, and acid mine drainage [19, 26, 27]. However, few acidophilic/tolerant SRB have been cultured [16, 26, 28–30]. A major potential advantage of using acidophilic sulfidogens would be to allow simpler engineering designs and reduce operational costs by using single on-line reactor vessels that could be used to both generate sulfide and selectively precipitate target metal(s). Precipitation and removal of many soluble transition metals, often present in AMD emanating from metal mines, may be achieved by ready biomineralization as their sulfides. The produced metal sulfides have different solubilities; therefore metals can be precipitated together or selectively by controlling concentrations of the key reactant S^2−^, which may be achieved by controlling pH (S^2−^ + H^+^  *↔* HS^−^). Copper sulfide, for example, is far less soluble than ferrous sulfide (respective log K_sp_ values of −35.9 and −18.8) and therefore CuS precipitates at pH 2, whereas FeS needs much higher pH to precipitate. Diez-Ercilla et al. [31] have also demonstrated that selective precipitation of metal sulfides occurs naturally in Cueva de la Mora pit lake (SW Spain) and the geochemical calculations match perfectly with the results of chemical and mineralogical composition. Ňancucheo and Johnson [3] showed that it was possible to selectively precipitate stable metal sulfides in inline reactor vessel testing two synthetic AMDs in acidic conditions (pH 2.2–4.8). In the first bioreactor, with a composition of feeding similar to AMD at the abandoned Cwm Rheidol lead-zinc mine in mid-Wales, zinc was efficiently precipitated (>99%) as sulfide inside the reactor while both aluminum and ferrous iron remain in solution (>99%) and were washed out of the reactor vessel. The second sulfidogenic bioreactor was challenged with a synthetic AMD based on that from Mynydd Parys, North Wales. Throughout the test period, all the copper present in the feed liquor was precipitated (confirmed as copper sulfide) within the bioreactor, but none of the ferrous iron was present in the solids. Although the initial pH at which the bioreactor was operated (from pH 3.6 to 2.5) caused some coprecipitation of zinc with the copper, by progressively lowering the bioreactor pH and the concentration of the electron donor in the influent liquor, it was possible to precipitate >99% of the copper within the bioreactor as CuS and to maintain >99% of the zinc, iron, and aluminum in solution. Glycerol was used as energy and carbon source (electron donor) and the generalized reaction is [1]

(1) 4C3H8O3+10H++7SO42−+Cu2++Zn2++Fe2+⟶12CO2+5H2S+CuS+ZnS+Fe2++16H2O

This low sulfidogenic bioreactor system was also demonstrated to be effective at processing complex acidic water draining from the Mauriden mine in Sweden [18]. Throughout the test period, zinc was removed from the synthetic mine water as ZnS, from which the metal could be recovered, as in the case at the Budel zinc refinery in The Netherlands [24]. Recently, Falagán et al. [32] have operated this sulfidogenic reactor to mediate the precipitation of aluminum in acidic mine waters as hydroxysulfate minerals. Besides, this bioreactor was tested to demonstrate the recovery of over 99% of the copper present in a synthetic mine water drained from a copper mine in Carajás in the State of Pará, Brazil [33]. The sulfidogenic system was also operated under different temperatures. Although there were large variations in rates of sulfate reduction measured at each temperature, the bioreactor operated effectively over a wide temperature range (30–45°C) which can have major advantages in some situations where temperatures are relatively high for example in mine sites located in northern Brazil and in other regions where high temperatures are observed. Therefore, there would be no requirements to have temperature control (heating or cooling) to preserve the integrity of the acidophilic SRB reactor [33]. The perceived advantages of this system are that there are simple engineering and relatively low operational cost. The system can be configured to optimize mine water remediation and metal recovery according to the nature of the mine water, which are the constraining factors in using active biological technologies to mitigate AMD.

Metalloids such as arsenic are a common constituent of mine waters. Battaglia-Brunet and colleagues [34] demonstrated that As (III) can be removed by precipitation as a sulfide. The results demonstrated the feasibility of continuous treatment of an acidic solution (pH 2.75–5) containing up to 100 mg As (V). Under this approach, As (V) was reduced to As (III) directly or indirectly (via H_2_S) by the SRB and orpiment (As_2_S_3_) generated within the bioreactor. In addition, this process was also observed to occur naturally in an acidic pit lake [31].

Recently, Florentino and colleagues [35] studied the microbiological suitability of using acidophilic sulfur reducing bacteria for metal recovery. These authors demonstrated that the* Desulfurella* strain TR1 was able to perform sulfur reduction to precipitate and recover metals such as copper from acidic waste water and mining water, without the need to neutralize the water before treatment. One drawback on the of use sulfur reducing microorganisms is that a suitable electron donor needs to be added for sulfate reduction. Even though sulfate is present in AMD, the additional cost of electron donors (such as glycerol) for sulfate reduction is higher than the cost of the combined addition of elemental sulfur and electron donors. Subsequently elemental sulfur as an electron acceptor can be more economically attractive for the application of biogenic sulfide technologies. On the other hand, cheaper electron donor such organic waste material may be used but their variable composition makes it less suitable for controlled high rate technologies. Besides, dead algal biomass can release organic products suitable to sustain the growth of SRB. Therefore, Diez-Ercilla et al. [31] have proposed that under controlled eutrophication it could be possible to decrease the metal concentrations in acidic mine pit lakes.

## 3. Microbiology in Remediating Acidic Mine Waters

Based on 16S rRNA sequence analysis, microorganisms that catalyze the dissimilatory reduction of sulfate to sulfide include representatives of five phylogenetic lineages of bacteria (Deltaproteobacteria, Clostridia, Nitrospirae, Thermodesulfobiaceae, and Thermodesulfobacteria) and two major subgroups (Crenarchaeota and Euryarchaeota) of the Archaea domain (Table 2 shows a summary of sulfidogenic microorganisms used for their main characteristics). SRB are highly diverse in terms of the range of organic compounds used as a carbon source and energy, though polymeric organic materials generally are not utilized directly by SRB [13]. In addition, some SRB can grow autotrophically using hydrogen as electron donor and fixing carbon dioxide, though others have requirement for organic carbon such as acetate, when growing on hydrogen. Besides, many SRB can also use electron acceptors other than sulfate for growth, such as sulfur, sulfite, thiosulfate, nitrate, arsenate, iron, or fumarate [78].

Most species of SRB that have been isolated from acidic mine waste such as* Desulfosarcina*,* Desulfococcus*,* Desulfovibrio,* and* Desulfomonile *are neutrophiles and are active at neutral pH [14, 25]. Besides, for a long time the accepted view was that sulfate reducing activity was limited to slightly acidic to near neutral pH explained by the existence of microniches of elevated pH around the bacteria [21, 31]. Attempts to isolate acidophilic or acid-tolerant strains of SRB (aSRB) have mostly been unsuccessful, until recently [79]. One of the reasons for the failure to isolate aSRB has been the use of organic acids such as lactate (carbon and energy source) which are toxic to many acidophiles. In acidic media, these compounds exist predominantly as nondissociated lipophilic molecules and, as such can transverse bacterial membranes, where they dissociate in the circumneutral internal cell cytoplasm, causing a disequilibrium and the influx of further undissociated acids, and acidification of the cytosol [80]. In contrast, glycerol can be used as carbon and energy source as it is uncharged at low pH. In addition, many SRB are incomplete substrate oxidizers, producing acetic acid as a product, enough to limit the growth of aSRB even at micromolar concentration. To circumvent this problem and for isolating aSRB, overlay plate can be used to remove acetic acid. This technique uses a double layer where the lower layer is inoculated with an active culture of* Acidocella (Ac.) aromatica* while the upper layer is not. Therefore, the heterotrophic acidophiles metabolize the small molecular weight compounds (such as acetic acid) that derive from acid hydrolysis of commonly used gelling agents such agar. The advantage of* Ac. aromatica* is its use of a limited range of organic donors and that it does not grow on yeast extract, glucose, glycerol, or many other small molecular weight organic compounds that are commonly metabolized by acidophilic heterotrophic microorganisms. Overlay plates are considered to be more versatile and efficient, particularly for isolating acidophilic sulfidogens from environmental samples, given that these microorganisms cannot completely metabolize the substrate [20]. Using this technique, aSRB and nonsulfidogens have been isolated from acidic sulfidogenic bioreactors. Two acidophilic sulfidogens (*Desulfosporosinus (D.) acididurans and *Peptococcaceae strain CEB3) and strain IR2 were all isolated from a low pH sulfidogenic bioreactor at different stages of operation, previously inoculated with an undefined microbial mat found at abandoned copper mine in Spain [3]. Although not yet fully characterized, Peptococcaceae CEB3 appears to be a more thermotolerant and acidophilic SRB that can oxidize glycerol to CO_2_ [33].

In addition,* D. acididurans* grew successfully together with* Ac. aromatica* in a pH controlled bioreactor, showing an example of microbial syntrophy where this heterotrophic bacterium converted acetic acid into CO_2_ and H_2_ [17].* D. acididurans* tolerates relatively high concentrations of aluminum and ferrous iron and can grow in a pH range of 3.8–7, with and optimum pH at 5.5. The temperature range for growth was 15–40°C with (optimum pH at 30°C), and it can use ferric iron nitrate, sulfate, elemental sulfur, and thiosulfate as electron acceptors [78].* D. acidophilus*, the second acidophilic SRB validly described [26] isolated from a sediment sample collected in a decantation pond receiving acid mine effluent (pH ~ 3.0), showed high tolerance to NaCl. SRB belonging to the genus* Desulfosporosinus* are known to thrive in low pH environments together with members of the closely related genus* Desulfitobacterium* which have also been detected in reactors operating at low pH. Interestingly,* Desulfitobacterium* is a genus with members that can use sulfite as electron acceptor, but not sulfate. Some bacteria, phylogenetically related to sulfur reducers, have been also detected in AMD bioreactors as well in natural acidic conditions [29].

## 4. Natural Attenuation for the Design of AMD Remediation Strategies

Natural remediation of metal pollutants generally involves the catalytic action of microbial activities that can accelerate the precipitation reaction of soluble toxic compounds resulting in their accumulation in precipitates [81]. Such information from natural systems can be useful for the design of engineered systems. Natural attenuation of transition metals in AMD has been described, for example, at the Carnoulès mine in France [81] and the Iberian Pyrite Belt (IPB) in Spain [10]. Rowe and colleagues [82] described in detail such process at a small site at the abandoned Cantareras copper mine, which is located in the Tharsis, mine district in the IPB. They reported that SRB other than* Desulfosporosinus* spp. were responsible for precipitating copper (as CuS) in a microbial mat found at the bottom layer and dissolved organic carbon (DOC) originated from photosynthetic and chemosynthetic primary producers serving as substrates for the aSRB. The pH of AMD obtained from this bottom layer was extremely acidic (pH < 3), and the dark grey coloration was due to the accumulation of copper sulfide, presumably as a result of biosulfidogenesis. No iron sulfides (e.g., hydrotroilite; FeS·*n*H_2_O) were detected, presumably due to the low pH of the mine water even at depth. Because the solubility product of CuS (log Ksp at 25°C is −35.9) is much lower than that of FeS (−18.8), this sulfide mineral precipitates in acidic waters whereas FeS does not.

Furthermore, Sánchez-Andrea and colleagues [83] described in detail the importance of sulfidogenic bacteria of the Tinto River sediments (Spain) and their role in attenuating acid mine drainage as an example of performing natural bioremediation. The results showed that, for attenuation in layers where sulfate reducing genera such as* Desulfosporosinus* and* Desulfurella* were abundant, pH was higher and redox potential and levels of dissolved metals and iron were lower. They suggested that sulfate reducers and the consequent precipitation of metals as sulfides biologically drive the attenuation of acid rock drainage. Lastly, the isolation and further understanding of anaerobic acidophiles in natural environments such as Cantareras and Rio Tinto have led to the proposal of new approaches to selectively precipitate toxic metals from AMD, turning a pollution problem into a potential source of metals [3, 83].

## 5. Concluding Remarks

Mining companies are increasing the extraction of mineral resources guided by a higher market demand, and also supported by productivity improvement resultant from advances on prospection and extraction technologies. Increased production consequently results in a higher generation of residues that is a global concern. The mining process has been significantly developed; however, pollution is still one of the main challenges of the mining industry and will require innovative management tools.

Given the fact that protecting aquatic and terrestrial ecosystems from pollutants generated from mine wastes is a major concern, new strategies must be employed such as the application of robust and empirically design bioreactors as part of an integrated system for remediation of acidic mine water and metal recovery. Using novel acidophilic and acid-tolerant sulfidogenic microorganisms that are the key components for bioremediation and knowledge about the microbial interactions that occur in extremely acidic, metal-rich environments will help in the development of new methods for bioremediation purposes.

## Figures and Tables

**Table 1:** Summary of the various types of treatment for AMD (compiled from Sahoo et al. [15], Gazea et al. [36], Trumm [37], Taylor et al. [38], RoyChowdhury et al. [39], Johnson and Hallberg [22], Skousen [40], Skousen et al. [41], and Seervi et al. [42]).

System type	Applicability	Support materials	Mechanisms	Limitation
*Biological*				
Aerobic wetland (AeW)	Moderate acidity, net alkaline mine drainage	Organic matter, soil, limestone gravel	Oxidation, hydrolysis, precipitation	Required longer detention time and huge surface area
Anaerobic wetland (AnW)	Net-acidic water with high Al, Fe and DO	Organic matter, such as compost, sawdust, hay, and limestone gravel,	Sulfate reduction, metal precipitate as sulfides, microbial generated alkalinity	Required long residence time
Vertical flow wetland (VFW)	Net-acidic water with high Al, Fe and DO	Limestone, organic matter	Sulfate and Fe reduction, acid neutralization	High capital cost, potential for armoring and plugging with hydroxides
Sulfate reducing bioreactor (SRB)	Small flows or to situations, very acidic and metal rich water	Organic substrate such as hay, alfalfa, sawdust, paper, woodchips, crushed limestone and compost or manure	Microbial sulfate reduction	High capital cost, extremely low pH severely impact the efficiency of S reducing bacteria
Pyrolusite limestone beds	Moderate pH and where majority of acidity is related to Mn	Limestone, organic substrate, aerobic microorganism	Hydrolysis of Mn	Not suitable for drainage which contains high Fe, high maintenance
Permeable reactive barriers (PRB)	Groundwater, low DO	Organic matter, limestone, zero valent iron	Sulfate reduction, sulfide precipitates, neutralization	
Iron-oxidizing bioreactor	Acidic water	Fe-oxidizing bacteria and archaea	Fe oxidation	
Phytoremediation	Any AMD-impacted sites	Metal tolerant plant species	Phytoextraction and phytostabilization	Success depends on the proper selection of the metal-hyperaccumulator plant

*Geochemical*				
Anoxic limestone drain (ALD)	Acidic water with low Al, Fe, DO	Limestone gravel, compacted soil	Limestone dissolution, raise pH, precipitation	Fe-oxide armoring limestone limit permeability and cause plugging
Alkalinity producing system (APS)	Acidic water	Organic matter, limestone	Anoxic condition, neutralization, precipitation	
Open limestone channel (OLC)	Required steep slopes, net-acidic water with high Al, Fe and DO	Limestone	Limestone dissolution, neutralization	Armoring or the coating of the limestone, large amount is needed, decreases the neutralizing capacity
Limestone leach bed (LLB)	Low pH and metal-free water	Limestone,	Limestone dissolution, neutralization	Armoring with Fe hydroxides
Steel-slag leach bed (SLB)	Highly acidic and metal-free water	Steel slag	Raise alkalinity, neutralization	Not suitable for metal-laden water
Limestone diversion wells (LDW)	Sites that offer a suitable topographical fall	Crushed limestone aggregate	Hydraulic force, hydrolysis, and neutralization	Required refilling with limestone every 2–4 weeks
Limestone sand	Streamflow water	Sand-sized limestone	neutralizing acid	Coating of limestone
Low-pH Fe oxidation channels	Shallow channels	Limestone or sandstone aggregate	Fe oxidation, adsorption and coprecipitation	It removes some Fe, but removal efficiency has not been determined
Sulfide passivation/microencapsulation	Pit wall faces, sulfide bearing wastes rocks piles	Inorganic coating: phosphate, silica, fly ash, limestone; organic coating: humic acid, lipids, polyethylene polyamine, alkoxysilanes, fatty acid, oxalic acid, catechol	Prevent sulfide oxidation by inorganic and organic coating	Long-term effectiveness is still in question, organic coating expensive
Electrochemical cover	Tailing/waste rock	Conductive steel mesh, cathode, metal anode	Reducing DO by electrochemical process	High capital cost of anodes, no information available on large scale application

*Physical*				
Dry cover	Sulfide bearing wastes rock piles	Fine-grained soil, organic materials, synthetic material (plastic liners), vegetation	Minimize oxidation by physical barrier, neutralization precipitates	Short term effectiveness
Wet cover	Sulfide wastes	Under water	Disposing waste under water anoxic conditions	Require rigorous engineering design, high maintenance
Gas redox and displacement system (GaRDS)	Underground mines	CO_2_ and CH_4_ gas	Gas mixtures physically displace O2	Its only feasible where partial or complete flooding is not feasible

**Table 2:** Isolated sulfidogenic microorganisms and their main characteristics.

Microorganism	Temperature (°C)	pH^a^	Carbon and electron source	Electron acceptor	Source	Reference
*Thermocladium modestius*	45–82 (75)	2.6–5.9 (4.0)	Glycogen, starch, proteins	Sulfur, thiosulfate, L-cysteine	Hot springs (water, mud), Japan	[43–45]
*Caldivirga maquilingensis*	70–90	2.3–6.4 (3.7–4.2)	Glycogen, beef extract peptone, tryptone, yeast extract	Sulfur, thiosulfate, L-cysteine	Hot springs (water, solfataric soil mud), Mt Maquiling, Philippines	[46]
*Archaeoglobus lithotrophicus*	nd	6.0	Acetate	Sulfate, L-cysteine	nd	[47, 48]
*Archaeoglobus veneficus*	nd	6.9	H_2_, acetate, formate, pyruvate, yeast extract, citrate, lactate, starch, peptone	Sulfite, thiosulfate	Walls of active black smoker at middle Atlantic Ridge	[44]
*Archaeoglobus profundus*	nd	4.5–7.5	H_2_, acetate, pyruvate, yeast extract, lactate, meat extract, peptone, crude oil with acetate	Sulfate, thiosulfate, sulfite	Deep sea hydrothermal system off Guaymas, Mexico	[49, 50]
*Archaeoglobus fulgidus*	60–75 (70)	5.5–7.5 (6,0)	H_2_, CO_2_, formate, formamide, D(−)- and L( + )-lactate, glucose, starch, calamine acids, peptone, gelatin, casein, meat extract, yeast extract	Sulfate, thiosulfate, sulfite	Marine hydrothermal system, Nerone, Italy	[49, 51]
*Thermodesulfatator indicus*	55–80 (70)	6.0–6.7 (6.25)	H_2_, CO_2_; stimulated by methanol, monomethylamine, glutamate, peptone, fumarate, tryptone, isobutyrate, 3-CH 3 butyrate, ethanol, propanol and low amounts of acetate.	Sulfate	Marine hydrothermal system, Central Indian Ridge	[52]
*Thermodesulfobacterium hydrogeniphilum*	50–80 (75)	6.3–6.8 (6.5)	H_2_, CO_2_; stimulated by acetate, fumarate, 3-methylbutyrate, glutamate, yeast extract, peptone or tryptone	Sulfate	Marine hydrothermal system, Guaymas Basin	[53]
*Thermodesulfobacterium commune*	41–83	6.0–8.0 (7.0)	H_2_, CO_2_, pyruvate, lactate	Sulfate, thiosulfate	Hot springs (water, sediment and mats) Yellowstone National Park, USA	[54, 55]
*Thermodesulfobacterium thermophilum*	nd	6.0–8.0 (7.0)	H_2_, CO_2_, pyruvate, lactate	Sulfate, thiosulfate	nd	[55]
*Thermodesulfobacterium hveragerdense*	75	4.5–7.0 (7.0)	H_2_, pyruvate, lactate	Sulfate, sulfite	Hot springs (microbial mats), Iceland	[56]
*Thermodesulfobium narugense*	69	4.0–6.0 (5.5–6.0)	H_2_, CO_2_	Sulfate, nitrate, thiosulfate	Hot springs (microbial mats), Japan	[57]
*Desulfotomaculum* spp. (30 species)	nd	2.3–5.5	H_2_, CO_2_, formate, some (organic acids; lipids; or monoaromatic hydrocarbons)	Sulfide, sulfur, thiosulfate, Acetate, some (Fe (III), Mn (IV), U (VI) or Cr (VI))	Subsurface environments, rice fields, mines, oil spills	[58–62]
*Desulfosporosinus meridiei*	10–37	6.1–7.5	H_2_, CO_2_, acetate, some (lactate, pyruvate, ethanol)	Sulfate, some (nitrate)	Groundwater contaminated with polycyclic aromatic hydrocarbons, in Swan Coastal Plain, Australia	[63]
*Desulfosporosinus youngii*	8–39 (32–35)	5.7–8.2 (7.0–7.3)	Beef extract, yeast extract, formate, succinate, lactate, pyruvate, ethanol and toluene	Fumarate, sulfate, sulfite, thiosulfate	Artificial wetland (sediment)	[64]
*Desulfosporosinus orientis*	37–48	6.0–6.5	H_2_, CO_2_, formate, lactate, pyruvate, malate, fumarate, succinate, methanol, ethanol, propanol, butanol, butyrate, valerate, palmitate	Sulfate, sulfite, thiosulfate, sulfur	nd	[65]
*Desulfosporomusa polytropa*	4–37	6.1–8.0	H_2_, CO_2_, formate, lactate, butyrate, several alcohols, organic acids, carbohydrates, some amino acids, choline, betaine	Sulfate, Fe(OH)_3_	Oligotrophic lake (sediment), German	[66]
*Thermodesulfovibrio yellowstonii*	41–83	6.0–8.0 (7.0)	H_2_, CO_2_, acetate, formate, lactate, pyruvate	Sulfate, thiosulfate, sulfite	Hot springs (water, sediment and mats) Yellowstone National Park, USA	[67]
*Thermodesulfovibrio islandicus*	55	4.5–7.0 (7.0)	H_2_, pyruvate, lactate, formate	Sulfate, nitrate	Bioreactor inoculated with hot springs (microbial mats) sample, Iceland	[56]
*Desulfohalobium *spp. (6 species)	nd	5.5–8.0 (6.5–7.0)	H_2_, lactate, ethanol, acetate	Sulfite	hypersaline environments	[68, 69]
*Desulfocaldus terraneus*	58	nd	H_2_, CO_2_, amino acids, proteinaceous substrates and organic acids, producing ethanol, acetate, propionate, isovalerate/2-methylbutyrate,	Cystine, sulfur, sulfate	Sea oil facilities, Alaksa	[70]
*Desulfomicrobium* spp. (4 species)	25–30	nd	H_2_, lactate, pyruvate, Ethanol, formate	Sulfate, sulfoxyanions	Anaerobic sediments (Freshwater, brackish, marine), anaerobic strata or overlying water, and in saturated mineral or organic deposits.	[54, 69]
*Desulfonatronovibrio hydrogenovorans*	37–40	9.0–10.2 (9.0–9.7)	H_2_, formate	Sulfate, sulfite, thiosulfate	Alkaline soda lakes (anaerobic)	[71]
*Desulfonatronum spp.* (3 species)	20–45 (37–45)	8.0–10.0 (9.0)	H2, formate, Yeast extract, ethanol, lactate	Sulfate, sulfite, thiosulfate	Alkaline soda lakes (anaerobic)	[72]
*Desulfovibrio* spp. (47 species)	25–44 (25–35)	nd	H_2_, CO_2_, acetate, lactate, carbohydrates,	Sulfate, nitrate	nd	[73]
*Desulfomonile* spp. (2 species)	30–30 (37)	6.5–7.8 (6.8–7.0)	H_2_, CO_2_, benzoate, pyruvate, organic carbon, halogens	Sulfate, sulfite, thiosulfate, sulfur, Fe (III), Nitrate, U (VI)	Sludge	[74]
Syntrophobacteraceae (8 genera)	31–60	7.0–7.5	H_2_, CO_2_, acetate, formate, lactate, pyruvate,	Sulfate, sulfite, thiosulfate	Sewage sludge, freshwater, brackish, marine sediment, marine hydrothermal vents, hot spring sediments	[73, 75]
*Desulfobacterium anilini*	30	6.9–7.5	H_2_, CO_2_, butyrate, higher fatty acids, other organic acids, alcohols	Sulfate, sulfite, thiosulfate	Freshwater, Brackish water, Marine, and Haloalkaline habitats	[76]
*Desulfarculus baarsii*	35–39	7.3	H_2_, CO_2_, butyrate, higher fatty acids, other organic acids, alcohols	Sulfate, sulfite, thiosulfate	Freshwater, Brackish water, Marine, and Haloalkaline habitats	[76]
*Desulfobacteraceae *(12 genera)	10–40	nd	H_2_, CO_2_, Long-chain fatty acids, Alcohols, Polar aromatic compounds, and in some cases even Aliphatic, aromatic hydrocarbons	Sulfate, sulfite, thiosulfate	Freshwater, Brackish water, Marine, and Haloalkaline habitats	[77]
*Desulfosporosinus acidophilus*	25–40	3.6–5.2 (5.2)	H_2_, lactate, pyruvate, glycerol, glucose and fructose	Sulfate	Sediment from an acid effluent pond	[26]
*Desulfosporosinus acididurans*	15–40	3.8–7.0 (5.5)	H_2_, formate, lactate, butyrate, fumarate, malate, pyruvate, glycerol, methanol, ethanol, yeast extract, xylose, glucose, fructose	Ferric iron, nitrate, sulfate, elemental sulfur, thiosulfate	White river draining from the Soufriere hills in Monserrat (pH 3.2)	[78]

^a^Values closed by parenthesis are considered optimal pH; nd: not informed by consulted reference.
